# Colon Microbiocenosis and Its Correction in Patients Receiving Programmed Hemodialysis

**DOI:** 10.17691/stm2020.12.5.07

**Published:** 2020-10-28

**Authors:** I.V. Belova, A.E. Khrulev, A.G. Tochilina, N.S. Khruleva, N.A. Lobanova, V.A. Zhirnov, S.B. Molodtsova, V.N. Lobanov, I.V. Solovieva

**Affiliations:** Leading Researcher, Laboratory of Human Microbiome and Means of Its Correction; Academician I.N. Blokhina Nizhny Novgorod Scientific Research Institute of Epidemiology and Microbiology of Rospotrebnadzor (Russian Federal Consumer Rights Protection and Human Health Control Service), 71 Malaya Yamskaya St., Nizhny Novgorod, 603950, Russia;; Associate Professor, Department of Nervous Diseases; Privolzhsky Research Medical University, 10/1 Minin and Pozharsky Square, Nizhny Novgorod, 603005, Russia;; Senior Researcher, Laboratory of Human Microbiome and Means of Its Correction; Academician I.N. Blokhina Nizhny Novgorod Scientific Research Institute of Epidemiology and Microbiology of Rospotrebnadzor (Russian Federal Consumer Rights Protection and Human Health Control Service), 71 Malaya Yamskaya St., Nizhny Novgorod, 603950, Russia;; Assistant, Department of Hospital Therapy and General Practice named after V.G. Vogralik; Privolzhsky Research Medical University, 10/1 Minin and Pozharsky Square, Nizhny Novgorod, 603005, Russia;; Chief Physician; FESFARM NN, 11A Vasenko St., Nizhny Novgorod, 603003, Russia; Researcher, Laboratory of Human Microbiome and Means of Its Correction; Academician I.N. Blokhina Nizhny Novgorod Scientific Research Institute of Epidemiology and Microbiology of Rospotrebnadzor (Russian Federal Consumer Rights Protection and Human Health Control Service), 71 Malaya Yamskaya St., Nizhny Novgorod, 603950, Russia;; Researcher, Laboratory of Human Microbiome and Means of Its Correction; Academician I.N. Blokhina Nizhny Novgorod Scientific Research Institute of Epidemiology and Microbiology of Rospotrebnadzor (Russian Federal Consumer Rights Protection and Human Health Control Service), 71 Malaya Yamskaya St., Nizhny Novgorod, 603950, Russia;; 6^th^-year Student, Medical Faculty; Privolzhsky Research Medical University, 10/1 Minin and Pozharsky Square, Nizhny Novgorod, 603005, Russia;; Leading Researcher, Head of the Laboratory of Human Microbiome and Means of Its Correction Academician I.N. Blokhina Nizhny Novgorod Scientific Research Institute of Epidemiology and Microbiology of Rospotrebnadzor (Russian Federal Consumer Rights Protection and Human Health Control Service), 71 Malaya Yamskaya St., Nizhny Novgorod, 603950, Russia;

**Keywords:** microbiocenosis, colon microbiota, chronic kidney disease, programmed hemodialysis, probiotics, synbiotics

## Abstract

**The aim of the investigation** was to study the species composition of colon microbiocenosis in patients with chronic kidney disease receiving programmed hemodialysis treatment and to evaluate the efficacy of its correction using a new immobilized synbiotic.

**Materials and Methods.:**

Samples of colon microbiota from 62 patients undergoing programmed hemodialysis were studied before and after a course of diet therapy that included probiotic components, in particular, the immobilized synbiotic LB-complex L. Isolation of microorganisms was carried out according to our original method; for bacteria identification, a MALDI-TOF Autoflex speed mass spectrometer (Bruker Daltonik, Germany) was used in the Biotyper program mode. The results were assessed using the criteria proposed by the authors and based on the OST 91500.11.0004-2003. The efficacy of the immobilized synbiotic was determined based on the clinical data, questionnaires, and bacteriological tests.

**Results.:**

In patients receiving programmed hemodialysis (before the start of the diet therapy), chronic moderate inflammation and azotemia were found. Dysbiotic changes in microbiocenosis were revealed in all the examined patients; in the absence or suppression of lacto- and bifidoflora, the number and diversity of *Bacteroides spp.*, *Clostridium spp.*, *Collinsella spp.*, *Eggerthella spp.* and other bacteria increased, which was consistent with the theory of functional redundancy of gut microbiota. From the answers to the questionnaires, a decrease in the quality of life was found (up to 70 points out of 100) according to six of the eight scales used. After the combined therapy using the synbiotic LB-complex L in the study group, 56% of the examined patients showed their microbiocenosis restored to normal; no grade III dysbiosis was detected in any patient. There was a significant decrease in CRP and ESR in these patients and an improvement in the quality of life by criteria reflecting physical health.

**Conclusion.:**

In patients receiving programmed hemodialysis, the addition of a probiotic component in the diet therapy restores the evolutionarily determined structure of the microbiocenosis, normalizes its functions, and leads to an overall improvement in health and quality of life.

## Introduction

Most patients at stage V of chronic kidney disease (CKD) that receive treatment with programmed hemodialysis (PH) have a variety of homeostatic disorders, leading to anemia, water and electrolyte imbalance, protein-energy deficiency, acidosis, insulin resistance, mineral and bone disorders, and dysbiosis. The number of such patients increases from year to year [1]. According to the register of the Russian Dialysis Society, as of December 31, 2018, there were about 55 thousand CKD patients on dialysis in the Russian Federation [2]; it is assumed that by the year 2040, this number will further increase [3].

Patients with end-stage CKD on renal support therapy have more pronounced changes in the intestinal microbiota as compared with patients at the pre-dialysis stage. In those, the greatest changes are found in patients receiving PH, which may be due to a number of factors. Among them, changes in hemodynamics of the digestive tract during the dialysis procedure, fluctuations in the concentrations of uremic toxins due to intermittent hemodialysis procedures, diet restrictions including a lack of dietary fiber, as well as the effect of medications such as antibiotics, iron preparations, and phosphate-binding drugs [4]. In addition, metabolic acidosis, swelling of the intestinal wall, and decreased motility of the colon contribute to the development of dysbiosis. Due to the large amount of nitrogen breakdown products in the colon, there is a growth of opportunistic bacteria that utilize urea, produce uricase, indole, and p-cresolforming enzymes. As a result, the amount of uremic toxins increases, aggravating the state of chronic uremic intoxication superimposed on a minimal residual kidney function. This condition aggravates the dysbiosis and the course of CKD [5, 6]. Intestinal dysbiosis is manifested not only by digestive disorders (diarrhea, intestinal discomfort, and bloating) and local manifestations (aerophagia, belching, nausea, chronic food urticaria, etc.) but also by systemic disorders arising from the translocation of gut bacteria through the intestinal barrier, which can lead to infectious complications (including sepsis) in patients on dialysis [7, 8].

Currently, in the clinical guidelines for the treatment of stage V CKD by hemodialysis and hemodiafiltration [9], there is no reference to the treatment of intestinal dysbiosis. However, the current guidelines of the Ministry of Health of the Russian Federation “Standards of Medical Nutrition” [10] (the section “Dietary therapy for diseases of the kidneys and urinary tract”) recommends using biologically active food supplements in the diet therapy as sources of vitamins, minerals, amino acids, dietary fiber, pre- and probiotics. In addition, the CKD outcomes could be improved by supplementing the patient’s diet with synbiotics [11, 12, et al.].

Based on the above, **the aim of the investigation** was to study the species composition of colon microbiocenosis in patients with chronic kidney disease receiving programmed hemodialysis treatment and to evaluate the efficacy of its correction using a new immobilized synbiotic.

## Materials and Methods

Patients with CKD were enrolled in this randomized, parallel-group, controlled clinical trial using a continuous sampling method. The study involved 62 patients (36 women and 26 men) undergoing treatment with PH for 1 year or more (at least 3 sessions per week and no less than 4 h/session and 720 min per week). The dialysis adequacy index based on the urea reduction ratio Kt/V was no less than 1.4. All patients were placed in the daytime inpatient ward; they received no antibiotics for 2 months or longer before the study. The study was conducted in accordance with the Declaration of Helsinki (2013) and approved by the Ethics Committee of the Privolzhsky Research Medical University. Informed consent was obtained from each participant.

The patients at the dialysis stage of CKD were divided into the main group and the comparison group matched sex and age. Basic therapy for patients in both groups included a high-protein diet and, if necessary, drug therapy: antihypertensive — β-blockers (bisoprolol), blockers of Ca-channel (amlodipine), blockers of imidazoline receptors (moxonidine) — and the lipid-lowering atorvastatin or rosuvastatin. If indicated, additional medications were used for anemia (erythropoietin α (or β) or methoxy polyethylene glycolepoetin-β), iron deficiency (iron (III) hydroxide sucrose complex), and bone mineral disorders; some patients received active metabolites of vitamin D (calcitriol, paricalcitol), calcimimetics (cinacalcet), phosphate-binding agents (β-iron (III) oxyhydroxide complex), and protein-energy supplements (keto analogs of amino acids) [13].

The main group consisted of 32 patients with CKD at the dialysis stage: there were 19 women (59%) and 13 men (41%) aged 38 to 65 years (mean age 57.1±7.9 years). Their dialysis treatment period varied from 12 to 123 months (40.6±29.8 months). They received the basic therapy and, as a probiotic, the novel immobilized synbiotic LB-complex L. The group of comparison included 30 patients (17 women (57%) and 13 men (43%)) aged 34 to 65 years (54.7±8.4 years) with comparable dialysis experience. They received the same basic therapy and a placebo instead of LB-complex L.

All patients were examined for their complaints, clinical and laboratory findings (routine blood test for biochemistry and cell counts); the dialysis adequacy index (Kt/V for urea) was calculated for each patient. Quality of life assessment was performed using a Russian-language version of the SF-36 questionnaire (Short form medical outcomes study) [14].

The study of the species composition of the intestinal microbiota and the assessment of the microbiocenosis was carried out using our original technique and the OST 91500.11.0004-2003 guidance “Patient management protocol. Intestinal dysbiosis” [15, 16].

For bacteria identification, a MALDI-TOF Autoflex speed mass spectrometer (Bruker Daltonik, Germany) was used in the Biotyper 4.1.80 program mode.

As a probiotic component of the diet therapy, we used our proprietary immobilized multistrain synbiotic LB-complex L (Certificate of State Registration — RU.77.99.88.003.E.002522.06.18) [17]. LB-complex L is recommended as a source of probiotic microorganisms (*bifidobacteria* and *lactobacilli*) and zeolites (enterosorbents) that increase the body’s non-specific resistance and have a detoxifying effect. The six strains comprising this synbiotic have been documented as safe and approved for use as part of medical immunobiological preparations. They have no genetically modified analogues and meet the requirements to probiotic strains [18, 19]; specifically, they are highly active against a wide range of pathogenic and opportunistic microorganisms, resistant to antibiotics (by the plasmid-independent mechanism), and resistant to the gastric juice and bile. Zeolites of the Holinsky mineral deposit, selected as a matrix for the immobilization of probiotic strains, are approved for medical use (Certificate of State Registration — KZ.16.01.78.003.E.004706.08.15). A unique property of clinoptilolites is their selective ion exchange ability: they supply missing macro-, micro-, and ultra-elements to the body and remove excessive elements from it. Zeolites have pronounced sorption properties, since the openwork of the crystal lattice creates a large adsorption volume; they do not break down and do not undergo changes in the human body [20].

**Statistical processing** was performed using the standard software packages Statistica 6.1 and Microsoft Excel 2007. Data are presented as the arithmetic means (M) and the standard errors of the means (m). If the data distribution was different from normal, nonparametric methods of analysis were used. The significance of differences was assessed using the Mann–Whitney test. Differences between independent groups were considered significant at p<0.05.

## Results and Discussion

At the beginning of this study, patients on PH were carefully examined for the clinical and laboratory parameters, the quality of life, and the colon microbiocenosis. In 82% of all patients, at least one of the gastrointestinal symptoms was found. Thus, dyspeptic symptoms (heaviness in the epigastrium, fullness, early satiety) were detected in 52% of patients, chronic constipation (stool once in 3 days, difficulty in defecating, fecal type 2 — according to the Bristol scale) — in 44%, abdominal discomfort — 31%, flatulence — 20%, belching — 11%, and chronic diarrhea — 7%.

Before using the synbiotic, we found similar values of most indicators in the two groups: a moderate increase in ESR (43.7±21.4 and 42.4±18.9 mm/h) and CRP (6.8±3.1 and 6.5±2.9 g/L), normal leukocyte counts ((6.7–6.8)**·**109/L), increased levels of blood urea (19.6–19.4 mmol/L) and creatinine (697.1–688.5 μmol/L); the results indicated chronic moderate inflammation and azotemia in all patients receiving PH. The Kt/V index for urea was 1.43±0.16 and 1.41±0.18 (study and control groups), which reflected the adequacy of the dialysis regimen.

**Figure 1:**
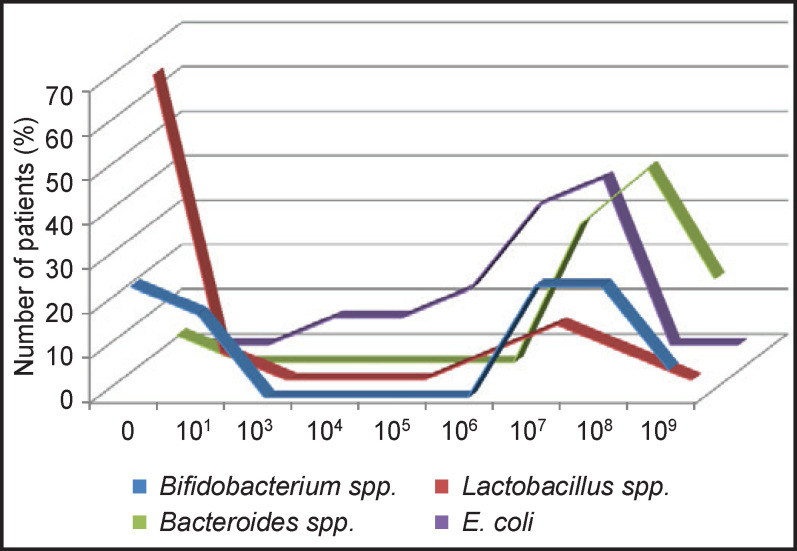
Quantitative characteristics of major components of the obligate intestinal microbiota in patients with chronic kidney disease receiving programmed hemodialysis

An in-depth study of the colon microbiota in patients with CKD on hemodialysis was conducted. Among the phylum *Actinibacteria*, bacteria of the genus *Bifidobacterium* were found in 75% of patients; in 43.7% of those, the number of bifidobacteria was significantly lower than normal (Figure 1).

**Figure 2:**
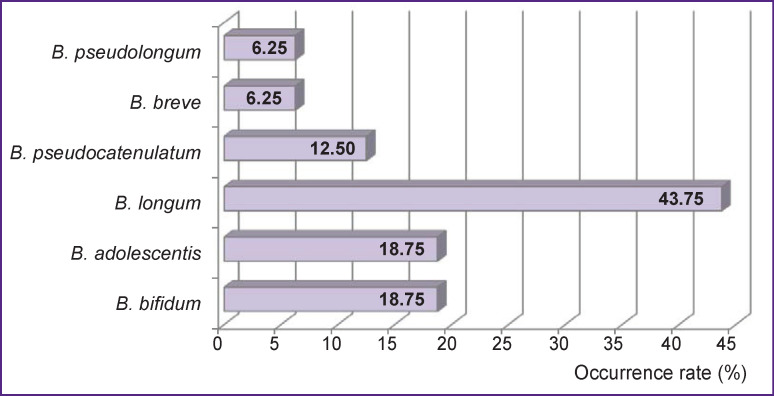
Occurrence rates for various species of the genus *Bifidobacterium* in patients with chronic kidney disease receiving programmed hemodialysis

One or two species of bifidobacteria were isolated from each patient, and *B. longum* predominated in these samples — 43.75% (Figure 2).

In addition to those shown in Figure 2, we also identified other species of this phylogenetic type. Thus, *Colinsella aerofaciens was* found in 31.25% of cases, *Eggertella lenta* — in 18.75%, and *Actinocorallia libanotica* — in just a few cases*.*

**Figure 3:**
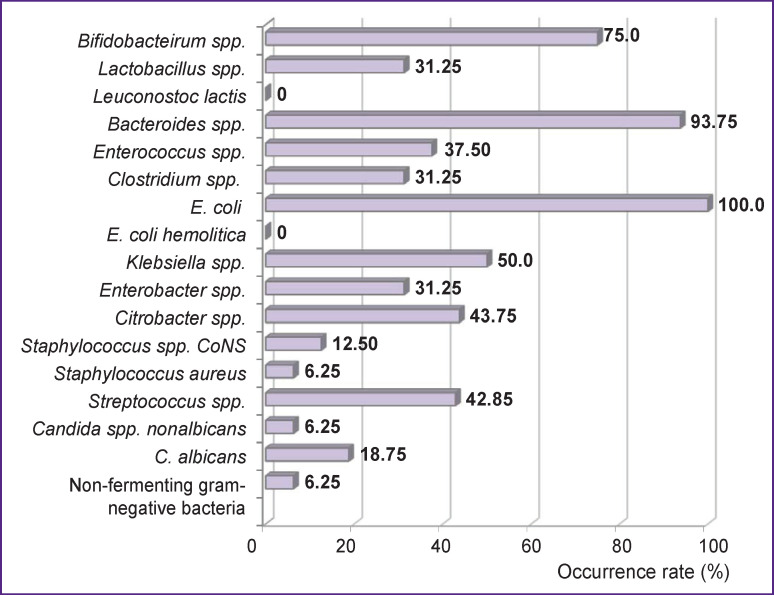
Occurrence rates for various species of the normal human colon microbiota in patients with chronic kidney disease receiving programmed hemodialysis

Of the phylum *Firmicutes*, microorganisms of the genus *Lactobacillus* were detected only in 31.25% of patients, while higher quantities (10^7^–10^8^ CFU/ml) were found in 18.75% of patients (Figure 3). Each patient contained 1–3 species of lactobacilli, most often *L. gasseri* (25%) and *L. rhamnosus* (12.5%). *L. salivarius*, *L. mucosae*, *L. ruminis*, *L. johnsonii* were identified in a few cases only.

Furthermore, the phylum *Firmicutes* was represented by bacteria of the genera *Eubacterium* (*E. limosum*), *Bacillus* (*B. acidicola*), *Clostridium spp.* (*C. innocuum* and *C. spiroforme*), *Staphylococcus spp.* (*S. aureus* and the group of coagulase-negative staphylococci *— S. epidermidis* and *S. warneri*), and microorganisms of the genus *Streptococcus* (*S. gallolyticus*, *S. lutetiensis*, *S. anginosus*, *S. salivarius*, and *S. parasanguini*).

**Figure 4:**
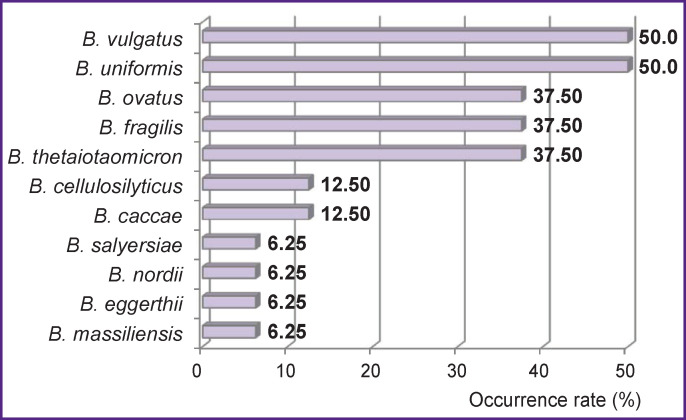
Occurrence rates for various species of the genus *Bacteroides spp.* in patients with chronic kidney disease receiving programmed hemodialysis

The phylum *Bacteroidetes* was represented by the genus *Parabacteroides* (*P. distansonis*) and *Bacteroides spp.* In a single patient, the colon microbiota might contain from 1 to 5 different species of bacteroides, mostly *B. vulgatus* (50%), *B. uniformis* (50%), *B. ovatus* (37.5%), *B. thetaiotaomicron* (37.5%), and *B. fragilis* (37.5%) (Figure 4)*.*

The phylum *Proteobacteria* was widely represented by various genera and species of the *Enterobacteriaceae* family, as well as microorganisms from the group of non-fermenting gram-negative bacteria (see Figure 3). Using our technique, we were able to isolate and characterize some rarely identified species of the family *Enterobacteriaceae*, like *Enterobacter asburiae*, *Enterobacter kobei*, *Citrobacter youngae*, *Serratia liquefaciens*, and *Raoultella planticola.*

**Figure 5:**
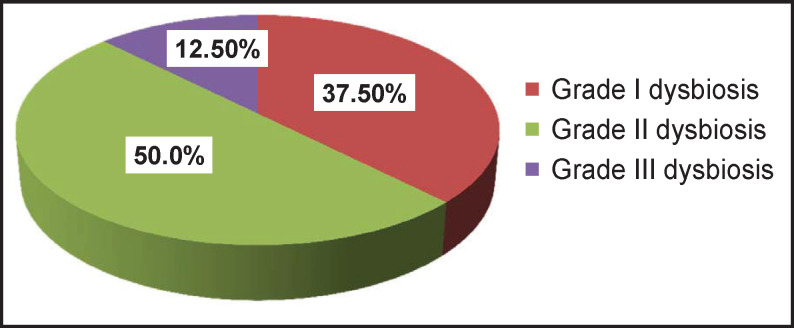
Dysbiotic disorders (grades I–III) of the colon microbiocenosis in patients with chronic kidney disease receiving programmed hemodialysis before starting treatment with synbiotic

Dysbiotic changes of varying degrees were detected in 100% of the examined patients (Figure 5).

Among the intestinal microbiota, the absence or suppression of lacto-and bifidoflora correlated with higher numbers and species diversity of *Bacteroides*, *Clostridium*, *Collinsella*, *Eggerthella*, and other microorganisms. This can be explained by the theory of functional redundancy of microbiota, which is widely covered in scientific literature [21]. Functional redundancy is an integral property of the intestinal microbiota, which characterizes the ability to perform similar metabolic functions by phylogenetically different microorganisms, i.e. the possibility of replacing some species with others without losing their functions. The concept of functional redundancy has been validated in metagenomic studies. For example, it has been demonstrated that the two major bacterial phyla *— Firmicutes* and *Bacteroides* — maintain stability in the intestinal ecosystem by using the functional redundancy, on the one hand, and the metabolic “specialization” (lactate and acetate production), on the other hand [22]. However, in CKD, due to the progression of renal failure, the abnormally high concentration of uremic toxins in the intra- and extracellular spaces stimulates their transport into the gastrointestinal tract. With the help of bacterial urease, urea rapidly converts into ammonium hydroxide that irritates the colon mucosa, which subsequently leads to inflammation. In the presence of bacteria, approximately 68% of creatinine transforms into creatine; the rest of creatinine is metabolized to 1-methylhydantoin, sarcosine, methylguanidine, etc. by predominating proteolytic bacteria (*B*. *fragilis*, *B. thetaiotaomicron*, *Enterobacter spp.*, and *Citrobacter spp.*). These metabolites — recognized as uremic toxins — contribute to the aggravation of renal failure [7].

Thus, under pathological conditions in humans, functional redundancy of microbiota can worsen the course of the disease [21]. As a result, restoration of microbiocenosis with the help of confirmed probiotic microorganisms (lactobacilli and bifidobacteria) is of particular importance: in the presence of these probiotics, proteolytic bacteria are removed from the gut thus reducing the production of uremic toxins. Then, the main metabolic function of these phyla (production of lactate, formate, and succinate) remains preserved.

After the combined therapy, both in the control and the LB-complex L treated group, bifidobacteria were detected in 100% of patients [17]. However, in the main group, the isolation procedure resulted in 10^9^–10^10^ CFU/g, while in control, the figure was 10^7^–10^8^ CFU/g (p<0.05). For lactobacilli, the respective values were 10^7^–10^8^ CFU/g in 100% of patients in the main group and 56.25% of patients from the comparison group. In the rest 43.75% of patients in control, lactobacilli were not found at all. Bacteroids (10^8^–10^9^ CFU/g) were detected in 100% of patients in the main group versus 10^6^–10^7^ CFU/g, in control (p<0.05).

After the treatment in the main group, the species composition of the anaerobic flora (*Bifidobacterium spp.*, *Collinsella spp.*, *Eggerthella spp.*, *Eubacterium spp.*, *Bacteroides spp.*, *Lactobacillus spp*., etc.) was more diverse than that in the group of comparison. This observation suggests that the anaerobic component of the colon microbiocenosis recovered in the main group to a fuller extent than in control. Among the aerobic bacteria after treatment, *E. coli* was detected in 100% of cases in both groups (10^6^–10^7^ CFU/g), while 25% of patients in the comparison group showed the presence of hemolytic forms of *E. coli*. In the main group, opportunistic microorganisms after treatment were detected with a lower frequency and in smaller numbers than those in the comparison group. Thus, *Klebsiella spp*. was found in 6.25% of patients in the main group versus 50.0% in the comparison group, *Citrobacter spp.* — in 12.5 and 56.25%, respectively*.* Next, *Enterobacter spp. w*ere not detected in any patient of the main group, in contrast to 25% of cases in the comparison group. *Staphylococcus spp.* bacteria were found in 6% (main) versus 18.8% (control). A similar trend was observed regarding other opportunistic microorganisms and fungi (*Raoultella spp.*, *Enterococcus spp.*, *Streptococcus spp.*, *Acinetobacter spp.*, *Corynebacterium spp.*, *Microbacterium spp.*, *Bacillus spp.*, *Candida spp.*, etc.).

**Figure 6:**
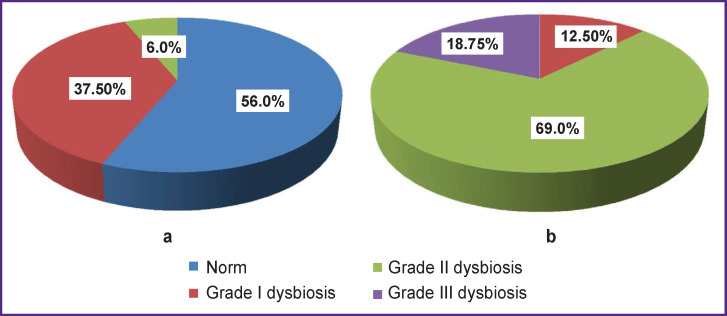
Dysbiotic disorders of colon microbiocenosis in patients with chronic kidney disease receiving programmed hemodialysis after treatment (a) main group; (b) comparison group

Thus, in 56% of patients in the main group, microbiocenosis recovered; grade III dysbiosis was not detected in any patient. In the comparison group, the microbiological picture worsened: there were more cases of pronounced dysbiosis of grades II and III (Figure 6).

After the treatment, patients in the main group reported improvements in their clinical symptoms: flatulence — 33% of patients, diarrhea — 50% of patients, constipation — 14% of patients, heaviness in the epigastrium, early satiety, and abdominal discomfort — 10% of patients. The occurrence of belching did not change. The above improvements in postprandial distress syndrome were associated with the normalization of intestinal motility including the gastric fundus and a reduction of endotoxemia and a better functioning of NO-dependent neurons involved in the autonomic innervation of the intestine. This mechanism was facilitated by the restoration of the evolutionarily determined microbiocenosis, the decrease in the number of proteolytic microorganisms, and the decrease in the toxic products resulted from taking the immobilized synbiotic. The elimination of toxins is mediated by the unique sorbent — zeolite, working as a carrier matrix in the structure of the synbiotic. The normalization of the species composition of the intestinal microbiocenosis also leads to a relief from chronic constipation by improving the motility of the colon and reducing the amount of uremic toxins. A reduction in chronic diarrhea, flatulence, and abdominal discomfort under the synbiotic treatment can be due to a decrease in the bacterial overgrowth syndrome, a decrease in the number of opportunistic microorganisms, and an increase in the number of lacto- and bifidobacteria.

In the main group, the levels of inflammation markers decreased after treatment: CRP (5.3 g/L) and ESR (36.2 mm/h).

The current concept of medical care requires not only restoration of the body function, but also normalization of patient’s wellbeing. Using the SF-36 questionnaire, we revealed an improved quality of life, specifically its physical component, in the main group. The most pronounced effect of the treatment was noted in the RP (role-physical) component: before treatment — 39.2, after — 45.1, in the P (pain intensity) scale: 65.2 and 71.3, respectively, and the GH (general health) scale: 52.6 and 58.8, respectively. Improvement of RP scores reflects changes in the physical condition and everyday activities of the examined patients. Positive dynamics of P scores reflects a decrease in pain in the study group. Better GH scores reflect positive dynamics in patient’s clinical condition and treatment prospects.

## Conclusion

This study provides new knowledge on species diversity and species representation of *Lactobacillus spp.*, *Bifidobacterium spp.*, *Bacteroides spp.*, *Eubacterium spp.*, *Clostridium spp.*, and other components of colon lumen microbiota in patients with chronic kidney disease who receive programmed dialysis. The addition of the immobilized multistrain synbiotic LB-complex L developed by these authors, in the diet therapy allows not only restoring the evolutionarily determined microbiocenosis, but also helps reducing inflammation and manifestations of postprandial distress syndrome, and improves the quality of life of patients.
